# Risk and prognostic significance of tuberculosis in patients from The TREAT Asia HIV Observational Database

**DOI:** 10.1186/1471-2334-9-46

**Published:** 2009-04-21

**Authors:** Jialun Zhou, Julian Elliott, Patrick CK Li, Poh Lian Lim, Sasisopin Kiertiburanakul, Nagalingeswaran Kumarasamy, Tuti Parwati Merati, Sanjay Pujari, Yi-Ming A Chen, Praphan Phanuphak, Saphonn Vonthanak, Thira Sirisanthana, Somnuek Sungkanuparph, Christopher KC Lee, Adeeba Kamarulzaman, Shinichi Oka, Fujie Zhang, Goa Tau, Rossana Ditangco

**Affiliations:** 1 National Centre in HIV Epidemiology and Clinical Research The University of New South Wales, Sydney, Australia; 2 Department of Medicine, Queen Elizabeth Hospital, Hong Kong, PR China; 3 Tan Tock Seng Hospital, Singapore; 4 Faculty of Medicine Ramathibodi Hospital, Mahidol University, Bangkok, Thailand; 5 YRG Centre for AIDS Research and Education, Chennai, India; 6 School of Medicine Udayana University & Sanglah Hospital, Denpasar, Bali, Indonesia; 7 Institute of Infectious Diseases, Pune, India; 8 Taipei Veterans General Hospital and AIDS Prevention and Research Centre, National Yang-Ming University, Taipei, Taiwan; 9 HIV-NAT/Thai Red Cross AIDS Research Centre, Bangkok, Thailand; 10 National Center for HIV/AIDS, Dermatology & STDs, Phnom Penh, Cambodia; 11 Research Institute for Health Sciences, Chiang Mai, Thailand; 12 Hospital Sungai Buloh, Kuala Lumpur, Malaysia; 13 University of Malaya, Kuala Lumpur, Malaysia; 14 International Medical Centre of Japan, Tokyo, Japan; 15 Beijing Ditan Hospital, Beijing, PR China; 16 Port Moresby General Hospital, Port Moresby, Papua New Guinea; 17 Research Institute for Tropical Medicine, Manila, the Philippines

## Abstract

**Background:**

To assess the risk and the prognostic significance of tuberculosis (TB) diagnosis in patients from The TREAT Asia HIV Observational Database, a multi-centre prospective cohort of HIV-infected patients receiving HIV care in the Asia-Pacific region.

**Methods:**

The risk of TB diagnosis after recruitment was assessed in patients with prospective follow-up. TB diagnosis was fitted as a time-dependent variable in assessing overall survival.

**Results:**

At baseline, 22% of patients were diagnosed with TB. TB incidence was 1.98 per 100 person-years during follow up, with predictors including younger age, lower recent CD4 count, duration of antiretroviral treatment, and living in high TB burden countries. Among 3279 patients during 6968 person-years, 142 died (2.04 per 100 person-years). Compared to patients with CDC category A or B illness only, mortality was marginally higher in patients with single Non-TB AIDS defining illness (ADI), or TB only (adjusted HR 1.35, p = 0.173) and highest in patients with multiple non-TB AIDS or both TB and other ADI (adjusted HR 2.21, p < 0.001).

**Conclusion:**

The risk of TB diagnosis was associated with increasing immunodeficiency and partly reduced by antiretroviral treatment. The prognosis of developing TB appeared to be similar to that following a diagnosis of other non-TB ADI.

## Background

The use of highly active antiretroviral therapy (HAART) has led to dramatic reductions in morbidity and mortality in HIV patients [1,2]. However, tuberculosis (TB) remains a common opportunistic infections and a major cause of death among patients with HIV, especially in sub-Saharan African and Asian countries [3-5], where there is a high background prevalence of TB [5-7].

The risk of TB in HIV-infected patients and the impact of TB diagnosis on disease progression in HIV infected patients have been well described in Africa [3,8-10]. The Asia-Pacific region has a large burden of both tuberculosis [7], with nearly 5 million prevalent cases and over 3 million new cases in 2006, and HIV, with an estimated 5 million people living with HIV and 380,000 new infections occurring in 2007 [11]. It is estimated that 2.5 million people are living with both infections in the region [5]. Despite the importance of these inter-related epidemics in the region, few studies have evaluated the risk of TB or its prognostic impact in patients with HIV [12-14].

Using data from The TREAT Asia HIV Observational Database (TAHOD), a multicentre prospective cohort study involving 17 clinical sites in the Asia-Pacific region, this paper aims to assess:

1) The risk of, and factors associated with, TB diagnosis among TAHOD patients with prospective follow up;

2) The prognostic significance of TB diagnosis on overall survival.

## Methods

TAHOD is a collaborative observational cohort study involving 17 sites in the Asia-Pacific region (See appendix). Detailed methods are published elsewhere [15]. Briefly, each site recruited 200 patients, including both patients on or not initiating HAART. Recruitment was based on a consecutive series of patients regularly attending a given site from a particular start-up time. Ethical approval for the study was obtained from the University of New South Wales Ethics Committee and respective local ethics committee.

The following data were collected: patient demographics and baseline data, CD4 and CD8 count, HIV RNA level, prior and new AIDS defining illness (ADI), date and cause of death, prior and current prescribed HAART, and reason for treatment change. Data are collected according to a common protocol. Upon recruitment, all available data prior to entry to TAHOD (considered as retrospective data) are extracted from patient case notes. Prospective data are updated six-monthly at each clinic and transferred to data management centre for aggregation and analyses. TAHOD sites are encouraged to contact patients who were not seen in the clinics in the previous 12 months.

TB diagnosis is defined as definitive when there is isolation (or culture) of *Mycobacterium tuberculosis*, *Mycobacterium bovis *or *Mycobacterium africanum *from a clinical specimen. TB is presumptively diagnosed when there is demonstration of acid-fast bacilli in a clinical specimen, or in a histopathological lesion when a culture is not available, in a person with signs or symptoms compatible with tuberculosis; or evidence of resolution of disease where treatment with two or more anti-tuberculosis medications have been prescribed and follow-up has been instigated.

Information on availability and regimen of TB treatment and prophylaxis were collected by surveying TAHOD sites. Anti-TB treatment is available to all HIV patients who are diagnosed with active TB in all participating TAHOD sites, with isoniazid, rifampicin, pyrazinamide and ethambutol given as intensive phase for at least 2 months, followed by isoniazid with either rifampicin or streptomycin or ethambutol as continuation phase for 4 to 10 months. TB prophylaxis is not available for patients at 13 of the 17 sites. For the 4 sites that provide prophylaxis, isoniazid is given for 6 to 12 months, to around less than 5% to 40% of the HIV patients, and is based on CD4 count (<200 cells/μL), a positive tuberculin skin test or recent close contact with another patient with active tuberculosis.

TAHOD sites were grouped into two categories, high and low/intermediate TB burden, according to WHO TB reports [16]. The high TB burden group included sites from China, Cambodia, India, Indonesia, the Philippines and Thailand. The low/intermediate burden group included sites from Hong Kong, Taiwan, Malaysia, Singapore and Japan.

Patients who had at least one prospective follow-up visit after entry to TAHOD were included. Baseline characteristics were compared between patients with and without a prior TB diagnosis (a TB diagnosis within 7 days after entry to TAHOD). Factors associated with baseline TB were assessed using univariate and multivariate logistic regression models.

The risk of TB among patients with prospective follow up was assessed using the person-time method. Follow-up was censored at date of TB diagnosis, or death, or the date of the most recent visit. Predictors of TB diagnosis were assessed using Cox proportional hazards models. ADI (including TB) diagnosed within 7 days after entry to TAHOD were considered as pre-existing events (prior diagnoses). The first diagnoses of TB during prospective follow up were included, which also included the prospectively diagnosed TB among patients with prior TB.

Overall survival after entry to TAHOD was also assessed using the person-time method. Follow-up was from entry to TAHOD until date of death or censored at date of the most recent visit. Death was reported using standardised cause of death form and independently reviewed by a clinician in the data coordinating centre. TB was separated from the list of other ADIs in this analysis since the aim was to compare the impact on disease progression of TB diagnosis with other ADIs. Patient's disease stage was updated when CDC category B illness, TB, or any other ADI was diagnosed during follow up. Baseline TB and incident TB were included as time-dependent variables in assessing overall survival. Disease stage was categorised as "CDC category A disease", "CDC category B disease", "TB only", "TB and CDC category B disease", "single non-TB ADI", "multiple non-TB ADIs" and "TB and other ADIs". Cox proportional hazards models were used to assess risk factors associated with TB diagnosis and overall survival.

In analyses of TB incidence and overall survival, antiretroviral treatment and CD4 counts were fitted as time dependent variables. The observations of each patient were updated at each time when there was a treatment change or a new CD4 count. Antiretroviral therapy was included in the model following the intention to treat principle, and was categorised according to the duration on treatment. We included the time-updated HIV viral load in the final survival model, the coefficient of disease stage in predicting survival, which is our primary interest, remains almost identical. Due to concerns about the missing data and collinearity, we decided not to include HIV viral load in the model.

Analysis was performed using the statistical package STATA (StataCorp LP 2007, STATA 10 for Windows, College Station, Texas 77845 USA). Predictors were assessed using multiple logistic or Cox proportional hazards models with a forward stepwise approach. The final multivariate model included covariates that remained significant at the 0.05 level (2-sided). Non-significant variables were also presented and adjusted for final multivariate models.

## Results

By June 2007, a total of 3516 patients had been recruited to TAHOD, including 3279 (93%) patients with at least one prospective follow up. The overall follow up rate in TAHOD is 85%. At entry to TAHOD, 759 (of 3516, 22% of all patients) had prior diagnosis of TB, among whom 717 (of 3279, 22%) had at least one prospective follow up. Up to 90% of the patients had at least one CD4 test during follow up and the median number of CD4 tests in each patient was 4 (IQR 2 to 7). However, just 58% of the patients had at least one HIV viral load test and the median number of RNA test was 4 (IQR 2 to 5). 830 patients were not on HAART at baseline and 412 of them started treatment during follow up. Most patients were initiated with combination treatment with at least two nucleoside reverse transcriptase inhibitors and one non-nucleoside reverse transcriptase inhibitors (73% of 2449 patients on treatment at baseline).

Baseline demographic characteristics are shown in Table 1. Patients with prior TB were less likely to be female, more likely of age between 31 to 40 years (compared to age less than 30 years), less likely to report HIV infection through homosexual contact or injecting drug use (compare to heterosexual contact), more likely to have lower CD4 counts and have no HIV viral load tests, and more likely to receive antiretroviral treatment and come from countries with high TB burden.

**Table 1:** Baseline characteristics associated with prior TB diagnosis

			Univariate		Multivariate	
			
Characteristics	No. patients	No.(%) with prior TB	OR	p-value	OR (95%CI)	p-value
**Total**	3279	717 (22%)				
**Sex**						
Male	2307	582 (25%)				
Female	972	135 (14%)	0.48	<0.001	0.38 (0.30, 0.47)	<0.001
**Age (years)**						
< = 30	783	141 (18%)				
31~40	1481	366 (25%)	1.49	<0.001	1.39 (1.10, 1.76)	0.006
41+	1015	210 (21%)	1.19	0.155	1.16 (0.89, 1.51)	0.261
**Reported mode of infection**						
Heterosexual contact	2288	584 (26%)				
Homosexual contact	614	66 (11%)	0.35	<0.001	0.47 (0.34, 0.63)	<0.001
Injecting drug use	132	34 (26%)	1.01	0.952	0.73 (0.47, 1.12)	0.154
Blood product	120	11 (9%)	0.29	<0.001	0.29 (0.15, 0.56)	<0.001
Other/unknown	125	22 (18%)	0.62	0.049	0.60 (0.37, 0.98)	0.042
**CD4 count at entry to TAHOD (cells/μL)**						
< = 50	241	84 (35%)				
51–100	209	78 (37%)	1.11	0.587	1.06 (0.74, 1.59)	0.780
101–200	524	142 (27%)	0.69	0.029	0.71 (0.50, 1.00)	0.054
201–300	578	121 (21%)	0.49	<0.001	0.58 (0.41, 0.83)	0.003
301+	1487	217 (15%)	0.32	<0.001	0.42 (0.30, 0.58)	<0.001
Not tested	240	75 (31%)	0.85	0.401	0.91 (0.61, 1.38)	0.665
**HIV viral load at entry to TAHOD (copies/mL)**						
< = 399	1133	188 (17%)				
400~10,000	188	24 (13%)	0.74	0.187	0.93 (0.58, 1.50)	0.774
10,001+	405	54 (13%)	0.77	0.123	0.92 (0.64, 1.32)	0.652
No. not tested	1553	451 (29%)	2.06	<0.001	1.36 (1.05, 1.76)	0.018
**Antiretroviral treatment at entry to TAHOD**						
No treatment	830	117 (14%)				
On treatment	2449	600 (25%)	1.98	<0.001	1.99 (1.56, 2.56)	<0.001
**Country of TB burden**						
High	1990	547 (27%)				
Low/intermediate	1289	170 (13%)	0.40	<0.001	0.55 (0.42, 0.70)	<0.001

OR: odds ratio. CI: confidence intervals.

Among the 3279 patients with at least one prospective follow up, 135 patients developed TB, giving a TB incidence of 1.98 per 100 person-years (95% confidence interval [CI] 1.67–2.34). CD4 count at time of TB diagnosis was available in 83 patients, with a median 159 cells/μL (IQR 50 – 261). Most of the TB diagnoses were definitive (92 of 135, 68%) or presumptive (30 of 135, 22%); there were 13 cases of possible TB (10%). However, no more information was collected in TAHOD regarding the availability of routine culture from the sites. Sites of TB infection were distributed as follows: pulmonary (60 of 135, 44%), extra-pulmonary (19 of 135, 14%), both pulmonary and extra-pulmonary TB (4 of 135, 3%) and unknown (52 of 135, 39%). The median TB incidence from the sites in low burden countries is 0.50 per 100 person years (IQR 0.46 to 0.57), while in high burden countries, 3.02 (IQR 0.98 to 4.85).

Factors associated with the rate of prospective TB diagnosis are presented in Table 2. In multivariate models, factors remaining included younger age, lower recent CD4 count, duration of antiretroviral treatment, and living in high TB burden countries. Patients aged more than 40 years had significantly lower rate of TB diagnosis than that of patients aged 30 or younger (adjusted HR 0,47, p = 0.003). A higher CD4 count was associated with a significantly lower rate of TB diagnosis. Compared to patients who were not receiving antiretroviral treatment, there was a significant increase in the rate of TB diagnosis within 90 days after initiating treatment (adjusted HR 4.01, p = < 0.001). The rate of TB diagnosis decreased continuously with the increase of duration of antiretroviral treatment. The rate of TB diagnosis was significantly lower among patients living in countries with low/intermediate TB burden than those in countries with high TB burden (adjusted HR 0.21, p = < 0.001). Although prior TB diagnosis was associated with higher rate of prospective TB (p = 0.038), it lost statistical significance after adjustments.

**Table 2:** Risk factors associated with TB diagnosis

					Univariate		Multivariate	
					
	No. of patients	Follow up (years)	No. of events	Rate	HR	p	HR (95% CI)	p-value
**Total**	3279	6815	135	1.98				
**Sex**								
Male	2307	4883	100	2.05				
Female	972	1932	35	1.81	0.88	0.510	0.77 (0.52, 1.14)	0.198
**Age at entry of TAHOD (years)**								
< = 30	1103	2203	56	2.54				
31~40	1401	2944	60	2.04	0.81	0.249	0.80 (0.56, 1.16)	0.237
41+	775	1668	19	1.14	0.45	0.003	0.47 (0.28, 0.79)	0.005
**Reported mode of infection**								
Heterosexual contact	2288	4896	102	2.08				
Homosexual contact	614	1243	16	1.29	0.62	0.074	1.29 (0.74, 2.23)	0.367
Injecting drug use	132	260	11	4.23	2.03	0.026	1.03 (0.54, 1.97)	0.917
Blood products	120	197	0	0.00	---	---	---	---
Other/unknown	125	219	6	2.74	1.29	0.544	0.94 (0.41, 2.15)	0.877
CDC classification for HIV infection *								
Category A	1747	3497	48	1.37				
Category B	622	1158	39	3.37	2.42	<0.001	1.33 (0.86, 2.05)	0.203
Category C	1020	2160	48	2.22	1.62	0.017	1.37 (0.89, 2.10)	0.150
CD4 count (cells/μL) *								
< = 50	313	251	27	10.74				
51–100	312	222	15	6.74	0.64	0.162	0.69 (0.36, 1.29)	0.243
101–200	928	842	30	3.56	0.34	<0.001	0.41 (0.24, 0.69)	0.001
201–300	1224	1250	23	1.84	0.17	<0.001	0.21 (0.12, 0.37)	<0.001
301+	2225	4197	30	0.71	0.07	<0.001	0.08 (0.05, 0.14)	<0.001
Not tested	68	53	10	18.98	1.83	0.106	1.02 (0.48, 2.15)	0.969
**Prior TB diagnosis**								
No	2562	5254	94	1.79				
Yes	717	1561	41	2.63	1.47	0.038	1.08 (0.74, 1.59)	0.684
Antiretroviral treatment *								
No treatment	830	985	40	4.06				
Treated, 0~90 days	453	83	14	16.91	4.01	<0.001	2.52 (1.31, 4.84)	0.006
Treated, 91–180 days	674	147	9	6.11	1.45	0.328	0.96 (0.45, 2.04)	0.909
Treated, 181–360 days	1017	414	5	1.21	0.30	0.013	0.24 (0.09, 0.62)	0.003
Treated 361+ days	2452	5186	57	1.29	0.30	<0.001	0.40 (0.26, 0.61)	<0.001
**Country of TB burden**								
High	1990	3644	115	3.16				
Low/intermediate	1289	3171	20	0.63	0.21	<0.001	0.28 (0.17, 0.45)	<0.001

Rate: per 100 person years. HR: hazard ratio. CI: confidence interval.

* Time dependent variable, patients can contribute person time to more than one category thus the sum of the categories may be more than the total number of patients.

A total of 142 deaths were recorded among 3279 patients during 6968 years of prospective follow up, a mortality rate of 2.04 per 100 person-years (95% CI 1.73–2.40). 69 deaths were reported to be directly related to HIV-associated illnesses (16 due to TB), 49 deaths due to other reasons, and cause of death was unknown for 24.

Factors associated with overall mortality are presented in Table 3. In multivariate analysis, mortality was higher in patients aged more than 40 years, with lower CD4 counts and who were not receiving antiretrovirals. Mortality also appeared to worsen when CDC category B illness, TB, or any other ADI was diagnosed during follow up. Compared to patients with CDC category A disease only, mortality was greatest in patients with multiple ADIs other than TB (adjusted HR 2.27, p = 0.007) and patients with both TB and at least one other ADI (adjusted HR 2.12, p = 0.004).

**Table 3:** Risk factors associated with overall survival

					Univariate		Multivariate	
					
	No. of patients	Follow up (years)	No. of events	Rate	HR	p	HR (95% CI)	p-value
**Total**	3279	6968	142	2.04				
**Sex**								
Male	2307	4992	105	2.10				
Female	972	1976	37	1.87	0.89	0.527	1.07 (0.73, 1.57)	0.732
**Age at entry of TAHOD (years)**								
< = 30	1103	2270	40	1.76				
31~40	1401	3016	50	1.66	0.95	0.812	0.86 (0.57, 1.32)	0.497
41+	775	1682	52	3.09	1.78	0.006	1.79 (1.18, 2.71)	0.006
**Reported mode of infection**								
Heterosexual contact	2288	5013	97	1.94				
Homosexual contact	614	1261	12	0.95	0.47	0.014	0.68 (0.21, 1.25)	0.368
Injecting drug use	132	276	14	5.08	2.54	0.001	1.40 (0.27, 2.53)	0.772
Blood products	120	196	7	3.58	1.77	0.144	1.57 (0.26, 3.44)	0.721
Other/unknown	125	222	12	5.40	2.65	0.001	1.63 (0.12, 3.01)	0.883
CD4 count (cells/μL) *								
< = 50	316	268	51	19.02				
51–100	325	246	16	6.49	0.35	<0.001	0.37 (0.21, 0.66)	0.001
101–200	939	875	20	2.29	0.12	<0.001	0.14 (0.08, 0.24)	<0.001
201–300	1238	1276	19	1.49	0.08	<0.001	0.10 (0.06, 0.17)	<0.001
301+	2242	4244	22	0.52	0.03	<0.001	0.04 (0.02, 0.07)	<0.001
Not tested	68	59	14	23.68	1.18	0.592	0.86 (0.46, 1.58)	0.622
**Country of TB burden**								
High	1990	3775	95	2.52				
Low/intermediate	1289	3193	47	1.47	0.62	0.007	0.86 (0.60, 1.26)	0.435
Antiretroviral treatment *								
No treatment	830	1006	44	4.37				
Treated, 0~90 days	475	88	8	9.14	1.89	0.103	1.08 (0.50, 2.35)	0.846
Treated, 91–180 days	703	154	5	3.24	0.67	0.405	0.39 (0.15, 0.99)	0.049
Treated, 181–360 days	1048	428	21	4.91	1.06	0.838	0.73 (0.42, 1.27)	0.265
Treated 361+ days	2478	5292	64	1.21	0.31	<0.001	0.32 (0.21, 0.51)	<0.001
Disease stage *								
CDC Category A	1444	2900	37	1.28				
CDC Category B	442	824	12	1.46	1.15	0.679	0.95 (0.49, 1.84)	0.886
TB only	328	634	13	2.05	1.65	0.122	1.53 (0.80, 2.91)	0.197
TB + CDC Category B	202	382	8	2.09	1.70	0.175	0.99 (0.45, 2.16)	0.976
Single non-TB ADI	523	1035	22	2.13	1.69	0.051	1.40 (0.80, 2.42)	0.237
Multiple non-TB ADIs	212	461	19	4.12	3.34	<0.001	2.27 (1.25, 4.10)	0.007
TB and other ADI(s)	336	732	31	4.23	3.57	<0.001	2.12 (1.27, 3.55)	0.004

Rate: per 100 person years. HR: hazard ratio. CI: confidence interval. ADI: AIDS defining illness.

* Time dependent variable, patients can contribute person time to more than one category thus the sum of the categories may be more than the total number of patients.

After fitting the Cox proportional hazards models, further testing of coefficients found that the mortalities were similar between patients with CDC category A and with B illness only (p = 0.433); among patients with TB only, with TB and CDC category B illness or with single non-TB ADI (p = 0.430); and between patients with multiple non-TB ADI and with TB and at least one other ADI (p = 0.410). Table 4 summarises the adjusted hazard ratios of these disease stages, initially separated, and then aggregated. Compared to patients with CDC category A or B illness only, mortality was marginally higher in patients with single Non-TB ADI, or TB only (adjusted HR 1.35, p = 0.173) and highest in patients with multiple non-TB AIDS or both TB and other ADI (adjusted HR 2.21, p < 0.001).

**Table 4:** Adjusted hazard ratios of disease stages on overall survival

Disease stage	Adjusted HR (95% CI)	p-value	Combined Disease stage	Adjusted HR (95% CI)	p-value
CDC Category A			CDC Category A or B		
CDC Category B	0.95 (0.49, 1.84)	0.886			

TB only	1.53 (0.80, 2.91)	0.197	TB only, or TB + CDC Category B, or Single non-TB ADI	1.35 (0.88, 2.07)	0.173
TB + CDC Category B	0.99 (0.45, 2.16)	0.976			
Single non-TB ADI	1.40 (0.80, 2.42)	0.237			

Multiple non-TB ADIs	2.27 (1.25, 4.10)	0.007	Multiple non-TB ADIs, or TB and other ADI(s)	2.21 (1.43, 3.42)	<0.001
TB and other ADI(s)	2.12 (1.27, 3.55)	0.004			

HR: hazard ratio. CI: confidence interval. ADI: AIDS defining illness.

Adjusted for age, CD4 count and antiretroviral treatments.

## Discussion

This study found a baseline prevalence of previous TB diagnosis of over 20% and a rate of prospective TB diagnosis during prospective follow up of 1.79 per 100 person-years. Prognosis following diagnosis of TB appeared to be no worse than following other ADI diagnoses.

We previously found [15] a higher prevalence of ADIs at study entry in TAHOD compared with cohorts in Western countries [17,18]. Much of the disparity in the prevalence of pre-existing ADI between TAHOD and western cohorts can be attributed to tuberculosis. [18-20]. In this analysis, patients from countries with high TB burden had a baseline prevalence of prior TB diagnosis of 27% and an incidence during follow up of 3.02 per 100 patient years, consistent with high TB burden countries in Asia [21] and Africa [9,22]. Although the prevalence of prior TB diagnosis in countries with low or intermediate TB burden was approximately half of that in countries with high TB burden countries in Asia, this risk was still higher than that seen in western cohorts [23,24].

During follow up the rate of prospective TB diagnosis was associated with a lower recent CD4 count, but not disease stages according to CDC classification. This discordance could partly be explained by the non directional nature of disease staging. Patients with category C disease treated with HAART have a reduced risk of TB, which reflected in the increase of CD4 count, but the disease stage does not change. Nevertheless, these data illustrate the ubiquity of TB risk across all stages of HIV infection.

Prior TB has been reported to increase the risk of subsequent TB [25]. In this study, however, prior TB lost statistical significance after adjustment, which is similar to findings from Lawn et al [10]. According to TAHOD sites, anti-TB treatment is available to all HIV patients who are diagnosed with active TB, which might provide at least time-limited reduction in the risk of TB in HIV patients [26].

Following HAART initiation, the rate of TB diagnosis was highest within 90 days after treatment initiation. Previously we have reported the increased rate of AIDS diagnosis shortly after HAART initiation in TAHOD [27]. It is probably due to immune reconstitution syndrome [28,29]; however, it is difficult in this observational setting to verify these cases with limited evidence collected. It could also be the fact that patients were generally more immune suppressed when HAART was initiated.

The rate of TB diagnosis decreased continuously with the increase of duration of antiretroviral treatment, but still above the estimated population incidence for the South-East Asian and Western Pacific regions [7]. This is consistent with the incomplete nature of TB-specific immune restoration [30] and emphasises the limitations of HAART in reversing the impact of the HIV epidemic on TB control [31]. HAART is usually commenced towards the end of a long period of increased TB risk [32] and although associated with a reduction in TB incidence, [3,21,23,24,33] this is off-set to some degree by increased survival and consequent longer period at risk. It is possible that further reductions in TB incidence during ART could be achieved through treatment of latent TB infection. Since only a small proportion of patients in TAHOD received isoniazid preventive therapy, its impact on the incidence of prospective TB diagnosis or interaction of effect with ART could not be assessed in this study.

In this cohort of patients with access to HAART when clinically indicated according to local treatment guidelines, there was a borderline significant increase in adjusted overall mortality, when comparing patients developing TB and no other ADI to those with no HIV-related illness. The adjusted hazard for death for patients developing TB only was similar to those developing other single AIDS defining illnesses, and when these groups were combined their risk of mortality was marginally different to patients with Category A or B disease. Similarly, in patients developing more than one ADI, TB seemed to carry a similar additional hazard of death as other ADIs. The data suggest that in a diverse population of people living with HIV in Asia with ready access to ART, the prognostic implication of active tuberculosis is more similar to other ADIs than CDC Category B disease. In individual patients, the prognostic utility of TB is limited by the wide spectrum of immunodeficiency present at the time of clinical presentation. Nevertheless, these data are consistent with consideration of TB as an indicator of a level of immunodeficiency for which initiation or change of ART is indicated in the absence of CD4 count.

A number of limitations should be considered when interpreting the above findings. Firstly, TAHOD is a newly established cohort with relatively small number of endpoints and prospective TB diagnosis. Thus statistical analyses have limited power to differentiate the prognostic significance of TB diagnosis, CDC stage B diagnosis and other ADIs. This also limited our ability to investigate the risk of TB by duration of HAART, HIV RNA level, treatment of latent TB or the prognostic significance of pulmonary versus extra-pulmonary disease. Secondly, TAHOD patients cannot be seen as entirely representative of HIV patients in the Asia-Pacific region as participating sites are generally urban referral centres. Thirdly, although the follow-up rate in TAHOD is satisfactory, ADI and other important events might be under-reported due to a patient not regularly visiting the clinics or if a patient dies. There may also be delay in reporting of AIDS diagnosis and death. And finally, CD4 counts and antiretroviral treatment are correlated; in particular, treatment leads to increases in CD4 count, has an impact on disease progression (CDC category) and so improves survival. The objective of this analysis was to estimate the role of TB on rate of death, and clearly treatment, CD4 count and CDC category are all confounders. The ARV treatment effects estimated from our models on the incidence of TB diagnosis and subsequent survival could indeed be biased as they are also adjusted for current and subsequent CD4 counts and CDC category.

However, the comparison of the effect of TB and other CDC category on survival should remain robust, and we believe should be adjusted for these factors. Specific modelling, such as marginal structural modelling [34] or G-estimation [35], which adjusts for the relationship between time-varying confounding factors and treatments, would need to be applied if the interest is to look at the effect of antiretroviral treatment on the development of TB and/or survival

## Conclusion

In conclusion, the diversity in rates of TB seen in TAHOD patients reflects the diversity in background population TB incidence seen across Asia-Pacific region. Risk of TB disease was also associated with increasing immunodeficiency and was only partly reduced by antiretroviral treatment. Following the diagnosis of TB, the prognosis appeared to be similar to that following a diagnosis of other AIDS defining illnesses. Competing interestsThe authors declare there are no competing interests.

## Authors' contributions

J Zhou participated in the design of the study, performed the statistical analysis, drafted and revised the manuscript. J Elliott participated in the design of the study, advised on the interpretation of results and the organisation of discussion, and comments on the manuscript. PCK Li, PL Lim, S Kiertiburanakul, N Kumarasamy, TP Merati and S Pujari participated in the design of the study, advised on the results and commented on the manuscript. YMA Chen, P Phanuphak, S Vonthanak, T Sirisanthana, S Sungkanuparph, CKC Lee, A Kamarulzaman, S Oka, FJ Zhang and G Tau contributed to the interpretation of the results and comments on the manuscript. R Ditangco conceived of the study, and participated in its design and coordination. All authors read and approved the final manuscript.

## Pre-publication history

The pre-publication history for this paper can be accessed here:

http://www.biomedcentral.com/1471-2334/9/46/prepub
